# Health literacy and knowledge of HIV and sexually transmitted infections among adolescents

**DOI:** 10.15649/cuidarte.5144

**Published:** 2026-08-20

**Authors:** Kimberly Alison Durand-Granados, Maricela Curisinche-Rojas

**Affiliations:** 1 Universidad Científica del Sur, Faculty of Nursing, Lima, Peru. E-mail: kimberlydurandgranados@gmail.com Universidad Científica del Sur Lima Peru kimberlydurandgranados@gmail.com; 2 Universidad Científica del Sur, Faculty of Nursing, GESPESS-Peru Research Group, Lima, Peru. E-mail: mcurisinche@cientifica.edu.pe Universidad Científica del Sur Lima Peru mcurisinche@cientifica.edu.pe

**Keywords:** Health Literacy, Knowledge, HIV, Sexually Transmitted Diseases, Adolescents, Alfabetización en Salud, Conocimiento, VIH, Enfermedades de Transmisión Sexual, Adolescentes, Letramento em Saúde, Conhecimento, HIV, Infecções Sexualmente Transmissíveis, Adolescentes

## Abstract

**Introduction:**

Inadequate knowledge of sexually transmitted infections (STIs) and the human immunodeficiency virus (HIV) places adolescents at increased risk for infection. Health literacy is an important strategy for reducing this risk.

**Objective:**

To determine the association between health literacy and knowledge of HIV and other STIs among adolescents.

**Materials and Methods:**

This analytical cross-sectional study included 204 adolescents from public schools. The Health Literacy Questionnaire and the HIV and other Sexually Transmitted Infections Knowledge Scale were used. Descriptive and bivariate analyses were performed using the chi-square test and Poisson regression with robust variance.

**Results:**

A total of 51.47% adolescents had inadequate knowledge, and 63.24% had inadequate health literacy. A significant association was found between the two variables (chi-square test, p = 0.009, 95% confidence interval [CI]). The crude prevalence ratio (cPR) was 1.44 (95% CI: 1.119-1.874), and the adjusted prevalence ratio (aPR) was 1.45 (95% CI: 1.123-1.880), adjusted for age, sex, and school grade level.

**Discussion:**

Health literacy equips individuals with the skills to access, understand, and use health information, improving knowledge for informed decision-making and the adoption of protective behaviors against HIV and STIs.

**Conclusion:**

Adolescents with adequate health literacy are more likely to have adequate knowledge of HIV and STIs. It is necessary to incorporate health literacy into health plans and promote information and education programs and interventions on these infections and comprehensive sexual health within and outside schools, using a multisectoral approach.

## Resumen

**Introducción:**

Los conocimientos inadecuados sobre infecciones de transmisión sexual (ITS) y el virus de la inmunodeficiencia humana (VIH) exponen a los adolescentes a un elevado riesgo de infección. La alfabetización en salud es una estrategia importante para reducir este riesgo.

**Objetivo:**

Determinar asociación entre alfabetización en salud y conocimientos sobre VIH y otras ITS en adolescentes.

**Materiales y métodos:**

Estudio transversal analítico, que consideró 204 adolescentes de colegios públicos. Se usaron el “Cuestionario de alfabetización en salud” y la “Escala de conocimientos sobre VIH y otras infecciones de transmisión sexual”. Se realizó análisis descriptivo y bivariado mediante Chi Cuadrado y Regresión de Poisson con varianza robusta.

**Resultados:**

51,47% de adolescentes presentó conocimiento inadecuado y 63,24% calificó con inadecuada alfabetización en salud. Se encontró asociación significativa entre ambas variables Chi2 (p = 0,009, IC 95%), RPc: 1,44 (IC 95%;1,119-1,874) y RPa: 1,45 (IC 95%: 1,123–1,880) ajustado por edad, sexo y grado escolar.

**Discusión:**

La alfabetización en salud provee capacidades para acceder, comprender y utilizar la información, que determina un mejor conocimiento para adoptar acertadas decisiones y acciones de protección frente a estas infecciones.

**Conclusiones:**

Los adolescentes con adecuada alfabetización en salud presentan una mayor probabilidad de tener conocimientos apropiados sobre VIH e infecciones sexuales. Es necesario agendar la alfabetización sanitaria en los planes de salud e impulsar programas e intervenciones de información y educación sobre estas infecciones y salud sexual integral dentro y fuera de los colegios con un enfoque multisectorial.

## Resumo

**Introdução:**

O conhecimento inadequado sobre infecções sexualmente transmissíveis (IST) e o vírus da imunodeficiência humana (HIV) expõe os adolescentes a um alto risco de infecção. A alfabetização em saúde é uma estratégia importante para reduzir esse risco.

**Objetivo:**

Determinar a associação entre alfabetização em saúde e conhecimento sobre HIV e outras IST em adolescentes.

**Materiais e métodos:**

Foi realizado um estudo transversal analítico com 204 adolescentes de escolas públicas. Foram utilizados o "Questionário de Alfabetização em Saúde" e a "Escala de Conhecimento sobre HIV e Outras Infecções Sexualmente Transmissíveis". Análises descritivas e bivariadas foram realizadas utilizando o teste qui-quadrado e regressão de Poisson com variância robusta.

**Resultados:**

51,47% dos adolescentes demonstraram conhecimento inadequado e 63,24% foram classificados como tendo alfabetização em saúde inadequada. Foi encontrada uma associação significativa entre ambas as variáveis (Qui-quadrado, p = 0,009, IC 95%), com uma razão de predição prévia (RPP) de 1,44 (IC 95%: 1,119–1,874) e uma razão de predição prévia (RPP) de 1,45 (IC 95%: 1,123–1,880), ajustada por idade, sexo e nível escolar.

**Discussão:**

O letramento em saúde proporciona a capacidade de acessar, compreender e utilizar informações, levando a um melhor conhecimento, tomada de decisões informadas e ações de proteção contra essas infecções.

**Conclusões:**

Adolescentes com letramento em saúde adequado têm maior probabilidade de possuir conhecimento apropriado sobre HIV e infecções sexualmente transmissíveis. É necessário incluir o letramento em saúde nos planos de saúde e promover programas e intervenções de informação e educação sobre essas infecções e saúde sexual abrangente, tanto dentro quanto fora das escolas, utilizando uma abordagem multissetorial.

## Introduction

Globally, the human immunodeficiency virus (HIV) and sexually transmitted infections (STIs) represent a significant burden of disease on health systems. In 2023, the World Health Organization estimated that 39.9 million people were living with HIV (PLHIV), 4.5 million people were infected each year with HIV and hepatitis B and C, and more than one million people acquired curable STIs every day^1^. Latin America accounts for 6% of PLHIV worldwide (2.3 million) and is among the three regions with increasing numbers of new infections^2^. In Peru, 110,000 PLHIV and 6,300 new infections were estimated for 2023^3^, with an upward trend over the past 10 years^4^.

Adolescents are a group vulnerable to HIV and STIs^5^ and developing interventions to promote their comprehensive health and well-being is a social commitment of the 2030 Agenda for Sustainable Development Goals^6^. In Latin America and the Caribbean (LAC), the population aged 10 to 19 is growing and represents 17% of the total population5, whereas in Peru it accounts for 15%^7^.

The number of adolescents living with HIV worldwide is increasing; in 2022, they accounted for 4% of all HIV infections and 10% of new infections^8^. In LAC, adolescents and young people represent 13% of PLHIV and account for one-third of new infections^5^. In Peru, they constitute 28% of PLHIV^4^ and STIs are also a major public health problem^9^. Various factors contribute to this vulnerability, including poverty, social exclusion, cultural factors, difficulties in accessing health services, early onset of sexual activity, and limited condom use, among others. Inadequate knowledge of HIV and STIs and the absence or scarcity of sexual health education, within and outside schools, are the main barriers to the prevention and control of these diseases^6^,^10^–^12^.

Studies from other countries highlight gaps in knowledge of HIV and STIs among adolescents, with differences based on gender and living context^13^,^14^. Two similar reports have been published in Peru^15^,^16^, although no publications were identified in Lima, the area with the highest burden of HIV cases in the country.

Insufficient or inaccurate knowledge exposes adolescents to risky attitudes and behaviors regarding HIV and STIs. Therefore, from a public health perspective, health literacy (HL) is essential during adolescence, a critical stage for learning and developing healthy habits and behaviors that will lead to informed decision-making and self-care empowerment^17^.

Various definitions of HL have been developed, and they all agree that HL refers to an individual’s ability to access, process, and apply health information to take appropriate action^17^. A systematic review reported a significant association between HL and health behaviors in adolescents, suggesting the need for future studies to better understand the influence of HL on their health-related decision-making^18^. In the Peruvian context, no studies on HL were identified in this specific population.

According to the integrated model of HL, core HL competencies are developed through consecutive steps of accessing, understanding, evaluating, and applying health information. These steps enable the acquisition or development of knowledge, skills, and motivation to act in health recovery, disease prevention, and health promotion^19^. In this context, an adequate level of HL could facilitate access to and understanding of information related to HIV and STIs, leading to greater knowledge of these diseases. Research reporting an association between HL and knowledge of HIV and STIs among adolescents is scarce^20^ and no reports were identified at the national level in Peru.

HL is becoming increasingly important in public health, making it necessary to gather information on its impact on knowledge of HIV and STIs to propose local interventions that help reduce the risks associated with these diseases. Therefore, the objective of this study was to determine the association between HL and knowledge of HIV and other STIs among adolescents.

## Materials and Methods

**Study design, setting, and participants**

This observational, cross-sectional, analytical study was conducted in two public secondary schools located in the southern part of Lima, in two districts with monetary poverty. Lima is the region with the highest concentration of HIV cases in Peru, accounting for 41% of all cases^3^. The study population consisted of 472 students enrolled in the fourth and fifth grades of secondary school at both institutions, distributed across 17 classes, with an average of 28 students per class.

A sample size of 212 students was estimated using the formula for finite-population proportions, with a 95% confidence level, a 5% margin of error and an expected proportion of 50% to maximize sample size. Participants were selected using stratified sampling; each class section constituted a stratum and a sample of 12 participants was estimated for each stratum, yielding a total of 204 participants.

The study included adolescents of both sexes aged 15 to 17 years who provided written assent and whose parents or legal guardians signed the informed consent form. Students who did not attend school on the day of the survey were excluded.

**Study variables**

The variable “knowledge of HIV and other STIs” was categorized as “adequate” when scores ranged from 13 to 24 (more than 50% of items answered correctly) and as “inadequate” when scores ranged from 0 to 12 (50% or fewer items answered correctly). Meanwhile, the variable “health literacy” was categorized as “inadequate” when scores ranged from 0 to 12 points and as “sufficient” when scores ranged from 13 to 16 points.

The covariates were age; sex (female and male); secondary grade (fourth and fifth grade); district of origin; “Have you received educational talks or counseling from healthcare personnel about HIV and STIs in the past 6 months?” (Yes/No); “Have you received information about HIV and STIs in the past 6 months through other sources such as the internet, television, brochures, or others?”(Yes/No);“Have you received educational talks, counseling, or guidance on HIV and STIs in the past 6 months from teachers, tutors, or another person at your school?” (Yes/No); “Have you received information on HIV and STIs in the past 6 months from friends and close family members?” (Yes/No); and “Are you aware that vaccines against human papillomavirus (HPV) and hepatitis B exist?” (Yes/No).

**Techniques and instruments**

The HIV and Other STIs Knowledge Scale for adolescents was used. This scale consists of 24 items organized into 5 dimensions (knowledge of HIV transmission, knowledge of other STIs, general knowledge of HIV, knowledge of condoms, and HIV prevention), each measured on a dichotomous scale (True/False). The instrument demonstrates acceptable internal consistency, with a Cronbach’s alpha of 0.88 and a test–retest correlation of 0.59 for the total item pool^21^.

Some terms were adapted to improve comprehension in the local context: “wet kiss” was replaced with “passionate kiss”; “HIV-positive person” with “person infected with HIV”; and “withdrawal method” with “coitus interruptus.” These modifications were intended to ensure semantic equivalence, and the construct’s clarity, relevance, and representativeness were validated by five nurses who are experts in the field, yielding an Aiken’s V coefficient of 0.95.

The Health Literacy Survey - European Questionnaire (HLS-EU-Q16) consists of 16 items that assess the perceived degree of difficulty in accessing/obtaining, understanding, processing/judging, and applying/ using information, rated on a dichotomous scale (very difficult/difficult and easy/very easy). This instrument has demonstrated high reliability (intraclass correlation coefficient: 0.923; kappa: 0.814) and high consistency as a unidimensional model with a Cronbach’s alpha of 0.982^22^.

Finally, a data collection form was developed for the study covariates.

**Data collection**

Data were collected between October and November 2024. Before data collection, the staff responsible for data collection received training in research ethics, confidentiality, HIV and STIs, and the standardized administration of the instruments, with an emphasis on communication with adolescents and the secure handling of information.

In coordination with school officials and teachers, in-person meetings were held with the adolescents to provide information about the study, answer questions, and invite them to participate. Interested students in each classroom were given the assent form to read and sign. They were then provided with an informed consent form to deliver to their parents or legal guardians to obtain signed authorization.

Subsequently, on scheduled dates and during school hours approved by the teachers, the informed consent forms signed by parents or legal guardians and the assent forms signed by the students were collected in each classroom. Instructions were then provided, the questionnaires were distributed, and the adolescents completed the surveys under the researcher’s supervision in approximately 20 minutes. The questionnaires were completed anonymously; no personally identifiable information was collected. Each participant’s questionnaire was assigned a unique code for database entry and organization.

**Data processing and analysis**

The data collected were entered into a Microsoft Excel 2019 spreadsheet, and statistical analyses were performed using the Statistical Package for Social Sciences (SPSS) version 25. A descriptive analysis of the study variables and covariates was conducted using absolute frequencies and percentages, and the results were presented in tables. Additionally, the mean age was calculated.

The prevalence of adequate and inadequate knowledge of HIV and STIs was estimated, as well as the prevalence of sufficient and inadequate HL. To determine associations or differences, bivariate analysis was performed using the chi-square test. Finally, to determine the strength of the association, prevalence ratios (PRs) were estimated using a Poisson regression model with robust variance, adjusted for the covariates of sex, age, and grade level at the secondary school. For all estimates, a statistical significance level of p<0.05 and a 95% confidence interval (CI) were used. The complete dataset is available for free access and consultation on Mendeley Data^23^.

**Ethical considerations**

This study was approved by the Institutional Research Ethics Committee of the Universidad Científica del Sur (Certificate No. 638-CIEI-Científica-2024). Written authorizations were obtained from the educational institutions, and ethical standards and principles for research involving human subjects were adhered to. Written informed assent was obtained from the adolescents, and written informed consent was obtained from their parents or legal guardians. The autonomy of adolescents and their parents was respected, and participants’ identities and data confidentiality were protected.

## Results

A total of 204 adolescents were surveyed. The mean age was 16.00 ± 0.72 years; 51.47% were found to have inadequate knowledge of HIV and STIs, and 63.24% had inadequate HL. Most participants reported having received information or guidance about HIV and STIs from healthcare personnel (60.78%), through sources such as the internet, television, brochures, or others (65.69%), and from teachers, tutors, or other personnel at their educational institution (69.12%), whereas only 49.51% reported receiving information from friends or family members Table 1.

**Table 1:** Characteristics, knowledge of human immunodeficiency virus and other sexually transmitted infections, and health literacy among adolescents from educational institutions in southern Lima (n=204)

Characteristics	% (n)
Age (years)	
15	21.08 (43)
16	46.57 (95)
17	32.35 (66)
Sex	
Female	48.53 (99)
Male	51.47 (105)
Location of the EI	
District 1	76.47 (156)
District 2	23.53 (48)
Secondary grade	
4th grade	52.94 (108)
5th grade	47.06 (96)
Received educational talks or counseling from healthcare staff about HIV and STIs	
Yes	60.78 (124)
No	39.22 (80)
Received information about HIV and STIs through the internet, television, brochures, or other sources.	
Yes	65.69 (134)
No	34.31(70)
Received educational talks, counseling, or guidance on HIV and STIs from teachers, tutors, or other people at their school	
Yes	69.12 (141)
No	30.88 (63)
Received information about HIV and STIs from friends and family members.	
Yes	49.51(101)
No	50.49 (103)
Is aware of the HPV and hepatitis B vaccines.	
Yes	55.88 (114)
No	44.12 (90)
Knowledge of HIV and other STIs	
Adequate	48.53 (99)
Inadequate	51.47 (105)
Health literacy	
Inadequate	63.24 (129)
Sufficient	36.76 (75)

EI: Educational Institution, HIV: Human Immunodeficiency Virus, STIs: Sexually Transmitted Infections, HPV: Human Papillomavirus.

Notable misconceptions about HIV and STIs included the belief that HIV is transmitted by sharing food or water (45.59%) or by kissing a person with HIV (47.06%); that syphilis is difficult to contract (42.65%); that treating the sexual partner of a person with gonorrhea is unnecessary (31.86%); and that the vaginal ring, intrauterine device (IUD), birth control pills, and withdrawal are effective in preventing HIV (49.02%, 25.00%, and 26.96%, respectively). In addition, 36.76% do not know the meaning of the window period Table 2.

**Table 2:** Knowledge of the human immunodeficiency virus and other sexually transmitted infections among adolescents from educational institutions in southern Lima (n=204)

Characteristics	False %(n)	True %(n)
Knowledge of HIV transmission		
HIV is transmitted through the air	96.57(197)	3.43 (7)
It is dangerous to share food or water with people infected with HIV.	54.41(111)	45.59 (93)
Washing the clothes of a person infected with HIV carries a risk of contracting the disease.	78.43(160)	21.57 (44)
Giving a passionate kiss to a person infected with HIV is a risk for HIV transmission.	52.94(108)	47.06 (96)
Hugging and kissing a person infected with HIV on the cheek carries a risk of HIV transmission.	80.88(165)	19.12 (39)
Knowledge of other STIs		
In most cases, gonorrhea usually clears up on its own.	77.45(158)	22.55 (46)
Syphilis is a disease that has practically disappeared.	68.14(139)	31.86 (65)
Syphilis can cause permanent damage, such as brain damage, blindness, or paralysis.	36.76(75)	63.24 (129)
Syphilis is currently very difficult to transmit.	57.35(117)	42.65 (87)
Hepatitis B never causes long-term complications.	73.53(150)	26.47 (54)
When a boy/girl has gonorrhea, it is not necessary to treat their partner.	68.14(139)	31.86 (65)
General knowledge of HIV		
AIDS is caused by a virus called "HIV".	23.53(48)	76.47 (156)
The main route of HIV transmission is through sexual intercourse.	15.69(32)	84.31 (172)
HIV is transmitted through sexual intercourse, blood transfusions, pregnancy, and breast milk.	13.73(28)	86.27 (176)
There is a risk of contracting HIV by sharing contaminated needles.	15.20(31)	84.80 (173)
HIV testing is usually performed through a blood test.	14.71(30)	85.29 (174)
An HIV-infected pregnant woman can transmit HIV to her baby.	21.57(44)	78.43 (160)
HIV affects the human immune system.	21.08(43)	78.92 (161)
The window period is the time it takes for the body to produce antibodies after HIV transmission.	36.76(75)	63.24 (129)
Knowledge about condoms		
Correct condom use is an effective method for preventing HIV transmission.	20.59(42)	79.41 (162)
Correct use of the female condom is just as effective as the male condom in preventing the transmis-sion of the AIDS virus.	28.43(58)	71.57 (146)
HIV Prevention		
The vaginal ring and the IUD are effective methods for preventing AIDS.	50.98(104)	49.02 (100)
Birth control pills are effective in preventing HIV transmission during sexual intercourse.	75.00(153)	25.00 (51)
The withdrawal method is a safe way to avoid the risk of HIV infection.	73.04(149)	26.96 (55)

HIV: Human Immunodeficiency Virus, STIs: Sexually Transmitted Infections, IUD: Intrauterine device, AIDS: Acquired Immunodeficiency Syndrome.

The findings on HL revealed that adolescents had limited skills or difficulty understanding what the doctor says (40.20%), finding information to address health problems (41.67%), considering information about health risks in the media to be reliable (37.25%), using information provided by the doctor to make decisions about their illness (32.84%), and deciding how to protect themselves from diseases based on information provided by the media (32.84%) Table 3.

**Table 3:** Health literacy among adolescents from educational institutions in southern Lima (n=204)

Characteristics	Very difficult / difficult %(n)	Very easy / easy %(n)
Finding information about treatments for the health conditions that concern you.	30.39(62)	69.61(142)
Finding out where to get professional help when you are sick.	26.47(54)	73.53(150)
Understanding what the doctor says.	40.20(82)	59.80(122)
Understanding the doctor's instructions on how to take prescribed medications.	26.96(55)	73.04(149)
Considering when a second opinion from another doctor may be needed.	31.37(64)	68.63(140)
Using information provided by the doctor to make decisions about your illness.	32.84(67)	67.16(137)
Following the doctor's instructions.	24.51(50)	75.49(154)
Finding information to address health issues.	41.67(85)	58.33(119)
Understanding health warnings related to habits such as smoking, lack of physical activity, or excessive alcohol consumption.	28.43(58)	71.57(146)
Understanding why you need to undergo disease screening tests or medical checkups.	32.35(66)	67.65(138)
Considering information about health risks reported in the media to be reliable.	37.25(76)	62.75(128)
Deciding how to protect yourself from diseases based on information provided by the media.	32.84(67)	67.16(137)
Finding activities that are good for your mental well-being.	30.88(63)	69.12(141)
Understanding health advice from family and friends.	21.08(43)	78.92(161)
Understanding information provided by the media on how to improve their health.	27.45(56)	72.55(148)
Recognizing which of your daily habits affect your health.	26.96(55)	73.04(149)

Bivariate analysis revealed a statistically significant association between HL and knowledge of HIV and STIs, with a p-value of 0.006 and a 95% CI Table 4.

**Table 4:** Association between health literacy and knowledge of the human immunodeficiency virus and other sexually transmitted infections among adolescents from educational institutions in southern Lima (n=204)

Characteristics	Knowledge of HIV and STIs		Total %(n)	p-value*
	Inadequate n= 99 %(n)	Adequate n= 105 %(n)		
Health Literacy				0.006
Inadequate	72.73(72)	54.29(57)	63.24(129)	
Sufficient	27.27(27)	45.71(48)	36.76(75)	
Age (years)				0.559
15	24.24(24)	18.10(19)	21.08(43)	
16	44.44(44)	48.57(51)	46.57(95)	
17	31.31(31)	33.33(35)	32.35(66)	
Sex				0.584
Female	49.49(49)	53.33(56)	51.47(105)	
Male	50.51(50)	46.67(49)	48.53(99)	
Secondary grade				0.117
4th grade	58.59(58)	47.62(50)	52.94(108)	
5th grade	41.41(41)	52.38(55)	47.06(96)	
Received educational talks or counseling from health personnel about HIV and STIs.				0.137
Yes	55.56(55)	65.71(69)	60.78(124)	
No	44.44(44)	34.29(36)	39.22(80)	
Received information about HIV and STIs through the internet, television, brochures, or other sources.				0.549
Yes	63.64(63)	67.62(71)	65.69(134)	
No	36.36(36)	32.38(34)	34.31(70)	
Received educational talks, counseling, or guidance on HIV and STIs from teachers, tutors, or another person at their educational institution				0.665
Yes	67.68(67)	70.48(74)	69.12(141)	
No	32.32(32)	29.52(31)	30.88(63)	
Received information about HIV and STIs from friends and family members.				0.782
Yes	50.51(50)	48.57(51)	49.51(101)	
No	49.49(49)	51.43(54)	50.49(103)	
Is aware of the human papillomavirus and hepatitis B vaccines.				0.223
Yes	51.52(51)	60.00(63)	55.88(114)	
No	48.48(48)	40.00(42)	44.12(90)	

HIV: Human Immunodeficiency Virus, STIs: Sexually Transmitted Infections, * Chi-square test.

The PR analysis showed that adolescents with sufficient HL were 44.80% more likely to have adequate knowledge of HIV and STIs than those with inadequate HL (95% CI: 1.119–1.874). In the analysis adjusted for the covariates of sex, age, and grade level, this statistically significant association was confirmed with a PR of 1.45 (95% CI: 1.123–1.880) Table 5.

**Table 5:** Association between health literacy and knowledge of human immunodeficiency virus and other sexually transmitted infections among adolescents, adjusted for study covariates (n=204)

Health literacy level	% (n)	Bivariate analysis: PR (95% CI)	p-value	Multivariate analysis: aPR* (95% CI)	p-value
Inadequate	63.24 (129)	1		1	
Sufficient	36.76 (75)	1.448 (1.119-1.874)	**0.005**	1.453 (1.123-1.880)	**0.004**

PR: Prevalence ratio, CI: Confidence interval. *aPR: Adjusted prevalence ratio estimated using a Poisson regression model with robust variance, adjusted for sex, age, and grade level.

## Discussion

The study highlights an association between HL and knowledge of HIV and STIs among adolescents from educational institutions in Peru, finding that adolescents with sufficient HL are more likely to have adequate knowledge of HIV and STIs. This positive association was maintained in the multivariate analysis adjusted for sex, age, and grade level. These results underscore the importance of greater attention to HL among adolescents, an extremely vulnerable population whose limited knowledge of HIV and STIs increases their risk of infection, especially within contexts of poverty and inequality.

Similar studies among adolescents are scarce both locally and globally. One study in Indonesia that, consistent with our results, reported that an HL intervention on HIV increased adolescents’ knowledge about the infection^20^. Two additional studies were conducted among adults and older adults in the United States and South Africa. In the first, a positive association was found between HL and knowledge about HIV/AIDS, even when adjusted for income level, education level, and risk perception score^24^. The South African study demonstrated that low HL is correlated with inadequate knowledge of HIV/AIDS, and that the use of appropriate educational methods can improve knowledge, increasing awareness of risk, prevention, and management. Similar to our findings, this study found significant associations with covariates such as age, sex, and education^25^. Therefore, these variables should be taken into account when planning interventions and designing HL and health education strategies.

The results obtained in the present study were consistent with the integrated theoretical model of HL, according to which adolescents with adequate HL would possess the skills not only to find and access health information, but also to evaluate, discern, and understand information received from various sources, thereby gaining an advantage and acquiring appropriate knowledge of HIV and STIs that will enable them to make better decisions regarding preventive measures and the adoption of healthy behaviors^19^. In the case of communicable infections such as HIV and STIs, this becomes particularly relevant and has a greater impact on public health, given that decisions and actions are focused on three areas of the health-disease continuum: i) seeking care within the healthcare system when infected or affected by HIV or an STI; ii) adopting preventive measures as individuals exposed to the risk of HIV and STIs; and iii) promoting healthy behaviors regarding HIV and STIs within and outside educational institutions and at home, as members of the community^19^.

Consequently, from a public health perspective and in the 21st century, it is necessary to promote and invest in HL among adolescents, with educational institutions, health services, and households serving as key settings for implementing HL programs through multisectoral collaboration and coordination (health, education, and other sectors). However, this represents a major challenge, considering that in countries like Peru, HL as such is a topic insufficiently known, understood, and addressed within these sectors, and even less so within households.

The present study found a predominance of adolescents with inadequate HL (63.24%), consistent with findings reported in previous studies. An exploratory review that included 82 studies found low to moderate levels of HL among adolescents and young adults^26^. Likewise, studies conducted in Spain and Taiwan reported a predominance of inadequate/insufficient or problematic levels of HL among adolescents, highlighting the need to pay greater attention to HL in this population, given their heightened vulnerability^27^,^28^.

Our findings on HL show that adolescents predominantly struggle to understand what their doctor explains, locate relevant information to address health problems, evaluate and distinguish reliable information about health risks disseminated in the media, use their doctor’s guidance to make decisions about their illness, and determine how to protect themselves from diseases based on information provided by the media. These findings reveal weaknesses and insufficient skills among adolescents in accessing, analyzing, understanding, and using health information for decisions related to their healthcare and the adoption of healthy behaviors.

This reality increases adolescents’ vulnerability to HIV, STIs, and other diseases, making it necessary for health and education authorities to prioritize and incorporate HL into their agendas. Currently, HL programs or interventions targeting adolescents are not explicitly included in health policies or plans; therefore, HL represents a complex goal to achieve without political and technical commitment.

The present results showed that more than half of the adolescents included in the study had inadequate knowledge of HIV and STIs. This is consistent with findings reported in various settings (Kenya, Southern Italy, Saudi Arabia, Iraq, and Peru), where low, inadequate, or insufficient knowledge was identified among adolescents, young people, key populations, and adolescent girls^15^,^29^-^32^. In line with our findings, these studies also identified friends, the internet, and social media as the main sources of sexual information among adolescents, whereas parents^30^,^31^ and teachers were cited less frequently. This reveals adolescents’ preference for inappropriate sources of information, which may contribute to the persistence of misconceptions about HIV and STIs.

The study identified that beliefs regarding HIV transmission through sharing food or water or kissing a person infected with HIV still persist, and that the vaginal ring or IUD, birth control pills, and withdrawal are effective measures for HIV prevention. Regarding STIs, it continues to be believed that syphilis is difficult to contract and that treating the sexual partner of a person with gonorrhea is unnecessary. These results highlight the need to strengthen health education in schools and to intensify educational programs and interventions on HIV and STIs, as well as age-appropriate, comprehensive sexual and reproductive health education with a gender-sensitive approach and adapted to the sociocultural context, in order to improve adolescents’ knowledge of risks and raise awareness regarding the adoption of preventive measures and healthy behaviors. In this regard, HL is an important strategy that contributes to increasing the impact of interventions. Adolescents with poor HL will have difficulty assessing, understanding, and using the information provided to them.

When analyzing the study results, the following limitations should be considered: i) the cross-sectional design of the study, which does not allow causal relationships between variables to be established; ii) participant sampling was not random, as the study included the first 12 students from each classroom who provided signed assent and had parental informed consent, which may limit the generalizability of the results; iii) response bias, as participants may not have answered the questions honestly; and vi) the lack of local or national research on HL and knowledge related to HIV and STIs limited the discussion and comparison of the findings with studies conducted in similar contexts.

Finally, as the first study in Peru to analyze this issue among adolescent school students, this study provides novel evidence positioning HL as a key determinant of sexual health knowledge. From a capabilities approach, these findings highlight the need to integrate HL as a structural component of comprehensive sexuality education policies and interventions aimed at developing skills to access, understand, evaluate, and use information that enables adolescents to make informed decisions about their sexual and reproductive health in a Latin American context where this variable has been scarcely explored in this group. These findings expand the analytical framework for HIV and STI prevention and provide input for the design of intersectoral interventions in the health and education sectors in Latin American countries or those with similar contexts.

## Conclusions

A statistically significant association was found between HL and knowledge of HIV and STIs among adolescents in educational institutions, indicating that adolescents with sufficient HL are more likely to have adequate knowledge of HIV and STIs. This positive association remained even after adjusting for variables such as sex, age, and grade level. There was evidence of a prevalence of inadequate HL and insufficient knowledge of HIV and STIs among adolescents, making it necessary to prioritize HL within health policies and plans and promote the implementation of information and education programs and interventions on these infections, sexuality, and comprehensive sexual health tailored to age, gender, and sociocultural contexts, both within and outside the school setting, using a multisectoral approach.
